# Plant elicitor peptide induces endocytosis of plasma membrane proteins in Arabidopsis

**DOI:** 10.3389/fpls.2023.1328250

**Published:** 2023-12-22

**Authors:** Yanping Jing, Xiaojiang Zheng, Rouhallah Sharifi, Jian Chen

**Affiliations:** ^1^ International Genome Center, Jiangsu University, Zhenjiang, China; ^2^ School of Life Sciences, Jiangsu University, Zhenjiang, China; ^3^ Chinese Education Ministry’s Key Laboratory of Western Resources and Modern Biotechnology, Key Laboratory of Biotechnology Shaanxi Province, College of Life Sciences, Northwest University, Xi’an, Shaanxi, China; ^4^ Department of Plant Protection, College of Agriculture and Natural Resources, Razi University, Kermanshah, Iran

**Keywords:** clathrin-mediated endocytosis (CME), trans-Golgi network/early endosome (TGN/EE), salicylic acid (SA), plant innate immunity, plant elicitor peptides (Peps)

## Abstract

In plants, the regulation of plasma membrane (PM) dynamics through endocytosis plays a crucial role in responding to external environmental cues and defending against pathogens. The *Arabidopsis* plant elicitor peptides (Peps), originating from precursor proteins called PROPEPs, have been implicated in various aspects of plant immunity. This study delves into the signaling pathway of Peps, particularly Pep1, and its effect on PM protein internalization. Using PIN2 and BRI1 as PM markers, we demonstrated that Pep1 stimulates the endocytosis of these PM-localized proteins through clathrin-mediated endocytosis (CME). CLC2 and CLC3, two light chains of clathrin, are vital for Pep1-induced PIN2-GFP and BRI1-GFP internalization.The internalized PIN2 and BRI1 are subsequently transported to the vacuole via the trans-Golgi network/early endosome (TGN/EE) and prevacuolar compartment (PVC) pathways. Intriguingly, salicylic acid (SA) negatively regulates the effect of Pep1 on PM endocytosis. This study sheds light on a previously unknown signaling pathway by which danger peptides like Pep1 influence PM dynamics, contributing to a deeper understanding of the function of plant elicitor peptide.

## Introduction

Plants, unlike animals, are rooted in place and cannot evade threats posed by pests and diseases. To counteract these external challenges, they have evolved highly conserved innate immune systems. In general, the plant innate immunity is triggered by the recognition of specific molecular components released by bacteria, fungi, or herbivores. Examples of these components include bacterial flagellin, elongation factor (EF)-Tu, fungal chitin, and peptidoglycans (Nürnberger et al., 2004; Zipfel and Felix, 2005; Boller and Felix, 2009; Macho and Zipfel, 2014). These molecules are categorized as MAMPs (microbe-associated molecular patterns), HAMPs (herbivore-associated molecular patterns), or VAMPs (virus-associated molecular patterns) based on their origin (Bartels and Boller, 2015). In addition, plants can release specific molecules known as damage- or danger-associated molecular patterns (DAMPs) in response to pathogen attacks or injuries, which play a regulatory role in plant immunity (Endo et al., 2014; Macho and Zipfel, 2014).

One well-studied DAMP in Arabidopsis is the plant elicitor peptides (Peps), originating from the C-terminal regions of precursor proteins called PROPEPs (Huffaker et al., 2006; Huffaker et al., 2013). *Arabidopsis* has eight PROPEPs, which generate eight small Pep peptides in response to pathogen invasion and physical injury (Huffaker et al., 2006; Bartels et al., 2013; Huffaker et al., 2013; Bartels and Boller, 2015; Klauser et al., 2015). Peps are recognized by two closely related receptors, PEPR1 and PEPR2, and initiate downstream signaling events, including the elevation of cytosolic Ca^2+^ levels, the generation of reactive oxygen species, the expression of defense-related genes, the formation of calluses, lignin deposition, and the regulation of root growth (Millet et al., 2010; Bartels et al., 2013; Beck et al., 2014; Ma et al., 2014; Jing et al., 2019; Jing et al., 2020; Jing et al., 2023).

Membrane proteins’ dynamics through endomembrane trafficking are crucial for plant growth and their ability to respond to environmental cues (Kleine-Vehn and Friml, 2008; Chen et al., 2011; Gu et al., 2017). Endomembrane trafficking encompasses several major pathways, including the biosynthetic secretory pathway, endocytic pathway, and vacuolar transport pathway (Aniento et al., 2022). The endoplasmic reticulum (ER)-Golgi-dependent biosynthetic secretory pathway plays a specialized role in providing and sorting plasma membrane and cell wall components (Sinclair et al., 2018). Endocytosis is the process by which cargo from the extracellular space or plasma membrane (PM) materials are internalized and redistributed into different subcellular destinations (Sorkin and von Zastrow, 2009; Aniento et al., 2022). Clathrin-mediated endocytosis (CME) is the primary mode of endocytosis that regulates the dynamics of PM proteins (Dhonukshe et al., 2007; Aniento et al., 2022). Accompanied by endocytosis, the internalized PM cargo is transported to the trans-Golgi network (TGN)/early endosome (TGN/EE) and then recycled back to the plasma membrane indirectly via recycling endosomes or recruited into intraluminal vesicles to finish the vacuolar transport pathway (Kaksonen and Roux, 2018; Mettlen et al., 2018; Aniento et al., 2022).

In our pursuit to unravel the signaling pathway of Peps in plants, our previous work highlighted Pep1’s role in stimulating the internalization of PM-located PIN-FORMED2 (PIN2) protein when externally applied (Jing et al., 2019). In the present investigation, we utilized two PM-localized proteins, PIN2 and BRASSINOSTEROID INSENSITIVE1 (BRI1), fused with GFP as markers of PM proteins to assess the effect of Pep1 on PM internalization. Our findings indicate that the application of exogenous Pep1 induces the endocytosis of PIN2-GFP and BRI1-GFP, a process mediated by the two clathrin light chains, CLC2 and CLC3. The internalized PIN2-GFP and BRI1-GFP accumulate in endosomes and are subsequently transported into the vacuole through the TGN/EE and PVC transport pathways. Interestingly, salicylic acid (SA) was found to negatively regulate the effect of Pep1 on PIN2 and BRI1 internalization. These discoveries unveil a novel signaling pathway through which Pep1 induces the endocytosis of PM-localized proteins.

## Results

### Pep1 induces endocytosis of plasma membrane-localized proteins

Previous investigations have documented the immunomodulatory effects of Peps and their role in inhibiting root growth in Arabidopsis (Zheng et al., 2018; Jing et al., 2019; Jing et al., 2020; Shen et al., 2020; Jing et al., 2023). Notably, treatment of roots with synthetic Pep1 led to a significant internalization of GFP-labeled PIN2 from the plasma membrane (PM). This observation raised the question of how Peps regulate the dynamics of PM proteins in terms of cellular internalization. To confirm the cellular internalization of PM induced by Pep1, we used the endocytic tracer Fei Mao dye 4-64 (FM4-64), a widely used marker in vesicle trafficking network research (Jelínková et al., 2010), to stain wild-type Columbia-0 (WT) seedlings. The dyed roots were incubated in half-strength MS liquid medium with or without 100 nM Pep1 for varying durations. As shown in **Supplementary Figure 1A**, the FM 4-64 labeled PM signals gradually diminished as intracellular puncta appeared (**Supplementary Figure 1**). Notably, the presence of Pep1 led to a significant increase in the density of intracellular puncta compared to the control condition, accompanied by a more pronounced reduction in PM fluorescence and a corresponding increase in intracellular space fluorescence (**Supplementary Figure 1**). These results suggest that Pep1 triggers PM endocytosis.

**Figure 1:**
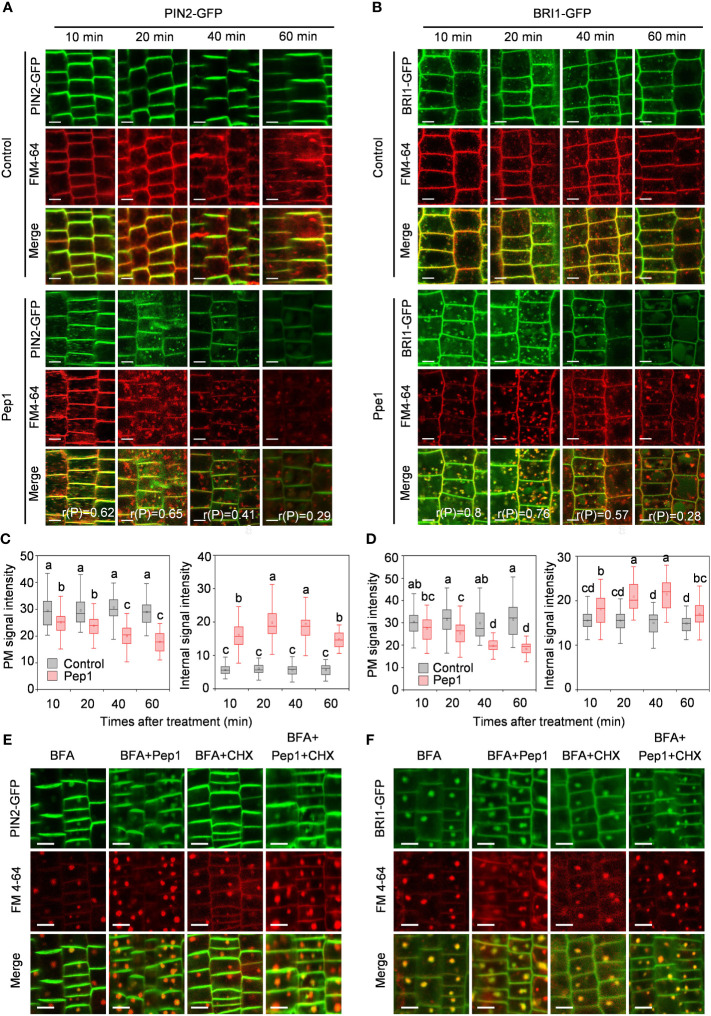
Pep1 induced the endocytosis of PIN2 and BRI1. **(A, B)** Pep1 promotes internalization of PIN2-GFP **(A)** and BRI1-GFP **(B)**. Six-day-old wild-type roots harboring PIN2-GFP **(A)** and BRI1-GFP **(B)** were stained in 2 μM FM4-64 solution for 5 min, rinsed three times, and incubated in half-strength MS liquid medium with or without (Control) 100 nM Pep1 as indicated for 10, 20, 40, and 60 min. The GFP and FM 4-64 fluorescence signals were collected. The 50 cells from 8 roots were analyzed in each of time point treatment, The experiments were repeated three times with similar results. r(P) represents the linear Pearson correlation coefficient indicates the percentage of signal overlap, and an r(P) value of 1.0 represents 100% colocalization. Bars=5 μm. **(C, D)** Quantitative analysis of plasma membrane and intracellular fluorescence intensity of PIN2-GFP **(C)** and BRI1-GFP **(D)** as in **(A, B)** (n= 60 cells from 8 roots per treatment). Boxs with different letters indicate significant differences as defined by two-way ANOVA with Tukey’s test (p < 0.05). **(E, F)** Pep1 promotes the BFA-visualized internalization of PM proteins. Six-day-old wild-type roots harboring PIN2-GFP **(E)** and BRI1-GFP **(F)** were stained in 2 μM FM4-64 solution for 5 min, rinsed three times, the roots were then treated with 25 μM BFA or 25 μM BFA cotreated with either 100 nM Pep1, 50 μM CHX or 100 nM Pep1 + 50 μM CHX for 60 min. The GFP and FM 4-64 fluorescence signals were collected. Bars=5 μm.

To further analyze the dynamics of PM proteins under Pep1 treatment, we explored the dynamics of PIN2-GFP and BRI1-GFP, two widely used PM protein markers in the endocytosis studies of membrane proteins (Dhonukshe et al., 2007). The signals of PIN2-GFP and BRI1-GFP were initially located on the PM of root epidermal cells (**Figures 1A, B**). However, when exposed to 100 nM Pep1, both PIN2-GFP and BRI1-GFP fluorescent signals appeared inside of the cell, forming intracellular puncta that colocalized with FM4-64 in wild-type roots (**Figures 1A, B**). The linear Pearson correlation coefficient (rP) for colocalization between PIN2-GFP and FM4-64 puncta exceeded 0.6, while for BRI1-GFP and FM4-64 puncta, it was above 0.7 after 10- and 20- minutes chase of Pep1 treatment. This indicates that both PIN2 and BRI1 undergo endocytosis following Pep1 treatment, in agreement with previous findings (Jing et al., 2019). Consequently, the PM-resident PIN2-GFP and BRI1-GFP gradually diminished, accompanied by an intensification of intracellular fluorescent signals (**Figures 1C, D**). After a 60-minute chase, PIN2-GFP and BRI1-GFP became concentrated in structures resembling vacuoles (**Figures 1A–D**), accompanied by decreased PM-localized fluorescent signals (**Figures 1A–D**). The intracellular fluorescent signals of PIN2-GFP and BRI1-GFP were increased at 20- and 40-minute chase of Pep1 treatment. However, at 60-minute chase, those intracellular fluorescent signals appeared to be decreased (**Figures 1A–D**).

The continuous endocytosis and recycling of proteins between the PM and the endomembrane system enable cells to adapt to extracellular stimuli (Kleine-Vehn and Friml, 2008; Chen et al., 2011; Gu et al., 2017). To investigate whether Pep1 treatment affects protein recycling, we used the fungal inhibitor brefeldin A (BFA), which blocks protein recycling by inhibiting BFA-sensitive ADP-ribosylation factor-guanine exchange factors (ARF-GEFs) (Naramoto et al., 2014). With BFA treatment, the fluorescent signals of PIN2-GFP and BRI1-GFP clearly accumulated in BFA bodies, which colocalized with FM4-64 signals (**Figures 1E, F**). Compared with the control condition, Pep1 treatment intensified a significant accumulation of PIN2-GFP and BRI1-GFP in BFA bodies (**Figures 1E, F**; **Supplementary Figure 2**). To determine whether these aggregated BFA bodies contained proteins from the PM or were newly synthesized, we used the protein synthesis inhibitor cycloheximide (CHX) for verification. CHX treatment partially inhibited the accumulation of PIN2-GFP and BRI1-GFP in BFA bodies induced by BFA (**Figures 1E, F**; **Supplementary Figures 2B, D**). However, when CHX was applied in combination with 100 nM Pep1, it failed to inhibit the formation of BFA bodies (**Figures 1E, F**; **Supplementary Figure 2**). This suggests that the accumulation of PIN2-GFP and BRI1-GFP in BFA bodies, induced by Pep1, primarily originates from the plasma membrane rather than newly synthesized proteins. In conclusion, these results demonstrate that Pep1 regulates PM dynamics by influencing the endocytosis of PM-localized proteins.

### Clathrin mediates Pep1-induced PM internalization

**Figure 2:**
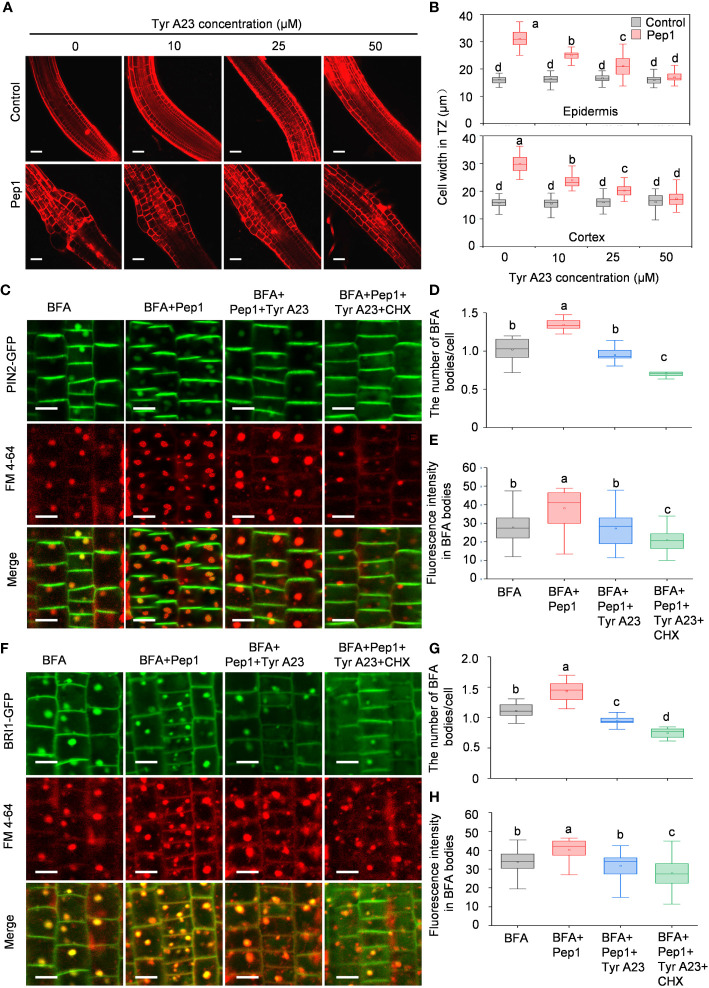
Tyr A23 interferes with the Pep1-induced internalization of PM proteins PIN2 and BRI1. **(A)** The cell swelling in the root transition zone. Five-day-old WT seedlings were transferred onto half-strength MS agar medium with various concentrations of Tyr A23 (ranging from 0 to 50 μM) supplemented with or without (control) 100 nM Pep1 for 12 h. The roots were stained with 5 uM propidium iodide (PI) for 15 s and photographed under a confocal laser-scanning microscope. The experiments were repeated three times with similar results. Bars = 100 um. **(B)** Quantitative analysis of epidermal and cortex cell width in TZ as in **(A)** (n= 30 cells from 6 roots per treatment). Boxs with different letters indicate significant differences as defined by two-way ANOVA with Tukey’s test (p < 0.05). **(C)** Evaluation of the BFA-visualized internalization of PIN2-GFP. Bars=5 μm. **(D)** Quantification of the BFA-visualized internalization of PM proteins in PIN2-GFP as in **(C)** (n= 50 cells from 8 roots per treatment). **(E)** Quantification of the PIN2-GFP fluorescence intensity in BFA bodies as in **(C)** (n= 100 cells from 10 roots per treatment). **(F)** Evaluation of the BFA-visualized internalization of BRI1-GFP. Bars=5 μm. **(G)** Quantification of the BFA-visualized internalization of PM proteins in BRI1-GFP as in **(F)** (n= 50 cells from 8 roots per treatment). **(H)** Quantification of the BRI1-GFP fluorescence intensity in BFA bodies as in **(F)** (n= 100 cells from 10 roots per treatment). Six-day-old seedlings were treated with 25 μM BFA or 25 μM BFA cotreated with either 100 nM Pep1, 100 nM Pep1 + 50μM Tyr A23 or 100 nM Pep1 + 50 μM Tyr A23 + 50 μM CHX for 60 min in **(C)** to **(H)**. In panels **(D, E, G, H)**, boxs with different letters indicate significant differences as defined by one-way ANOVA with Tukey’s test (p < 0.05).

Clathrin-mediated endocytosis (CME) is the predominant pathway responsible for regulating PM dynamics (Dhonukshe et al., 2007; Aniento et al., 2022). To gain a deeper understanding of the mechanism behind PM protein internalization induced by Pep1, we investigated the endocytosis of PIN2-GFP and BRI1-GFP using Tyrphostin A23 (Tyr A23), a well-established inhibitor that is widely used to interfere with the interaction of the tetrapeptide Yxxφ motif with the clathrin medium chain (Banbury et al., 2003; Xing et al., 2022). Before examining endocytosis, we noted structural changes in the root transition zone (TZ) following Pep1 treatment compared with normal growth, where the epidermis and cortex cells in the TZ experienced swelling (**Figures 2A, B**), consistent with our prior findings (Jing et al., 2019). We co-treated WT roots with a range of Tyr A23 concentrations (from 10 to 50 μM) alongside 100 nM Pep1. Interestingly, Tyr A23 treatment significantly mitigated the Pep1-induced cell swelling, with 50 μM Tyr A23 significantly abolishing the Pep1-induced cell swelling (**Figures 2A, B**). Furthermore, we analyzed the internalization of PIN2-GFP and BRI1-GFP. Clearly, 50 μM Tyr A23 effectively blocked the Pep1-induced gathering of PIN2-GFP and BRI1-GFP fluorescent signals into BFA bodies (**Figures 2C–H**), indicating the essential role of CME in regulating Pep1 signaling.

**Figure 3:**
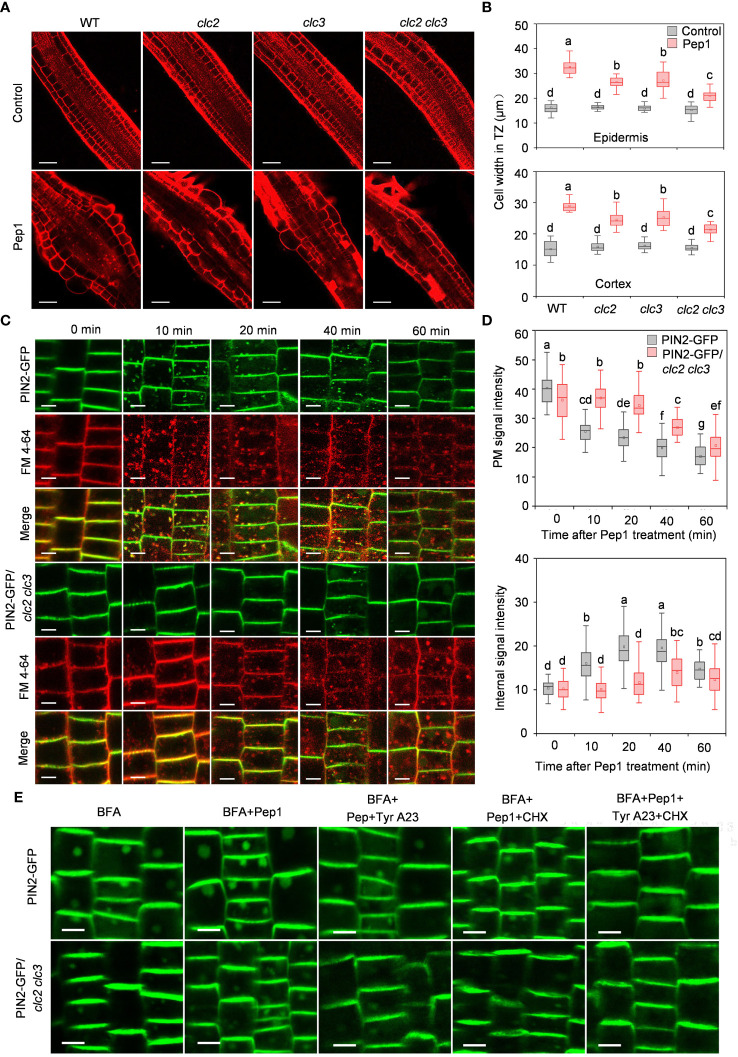
Clathrin dependence of Pep1-induced responses. **(A)** The cell swelling in the root transition zone. Five-day-old WT, *clc2*, *clc3* and *clc2 clc3* seedlings were transferred onto half-strength MS agar medium supplemented with or without (control) 100 nM Pep1 for 12 h. The roots were stained with 5 uM propidium iodide (PI) for 15 s and photographed under a confocal laser-scanning microscope. The experiments were repeated three times with similar results. Bars = 100 um. **(B)** Quantitative analysis of epidermal and cortex cell width in TZ as in **(A)** (n= 30 cells from 6 roots per treatment). Boxs with different letters indicate significant differences as defined by two-way ANOVA with Tukey’s test (p < 0.05). **(C)** The internalization of PIN2-GFP in WT and *clc2 clc3 mutant.* The roots of 6-day-old plants were stained in 2 μM FM4-64 solution for 5 min, rinsed three times, and incubated in half-strength MS liquid medium with 100 nM Pep1 as indicated for 10, 20, 40, and 60 min. The experiments were repeated three times with similar results. Bars=5 μm. **(D)** Quantitative analysis of plasma membrane and intracellular fluorescence intensity of PIN2-GFP in WT and *clc2 clc3 mutant* as in **(C)** (n= 60 cells from 8 roots per treatment). Boxs with different letters indicate significant differences as defined by two-way ANOVA with Tukey’s test (p < 0.05). **(E)** Evaluation of the BFA-visualized internalization of PIN2-GFP in WT and *clc2 clc3 mutant.* Six-day-old seedlings were treated with 25 μM BFA or 25 μM BFA cotreated with either 100 nM Pep1, 100 nM Pep1 + 50 μM Tyr A23, 100 nM Pep1 + 50 μM CHX or 100 nM Pep1 + 50 μM Tyr A23 + 50 μM CHX for 60 min. Bars=5 μm.

To delve further into the role of CME in controlling the Pep1 induced PM internalization, we examined Pep1 responses in clathrin light chains (CLCs) knockout mutants (*clc2*, *clc3*, and *clc2 clc3* double mutant) (Wang et al., 2013). Under Pep1 treatment, cell swelling was significantly reduced in *clc2* and *clc3* mutants compared with the wild-type plant (**Figures 3A, B**). Simultaneous mutation of *CLC2* and *CLC3* in the *clc2 clc3* double mutant further suppressed the Pep1-induced cell swelling (**Figures 3A, B**). Moreover, the application of 50 μM Tyr A23 completely blocked the Pep1 effect in the *clc2 clc3* double mutant (**Supplementary Figure 3**), suggesting that other clathrin proteins function redundantly with CLC2 and CLC3 in the regulation of Pep1 signaling. We proceeded to analyze PM internalization in the *clc2 clc3* double mutant continuously. The PIN2-GFP-labeled intracellular puncta and BFA bodies were significantly reduced in the *clc2 clc3* double mutant compared to those in WT roots under Pep1 treatment (**Figures 3C–E**; **Supplementary Figure 4**). The PM-resident PIN2-GFP in *clc2 clc3* double mutant was diminished and the intracellular fluorescent signals was increased at 40-minutes chase of Pep1 treatment, whereas the signal was delayed compared with these in WT plant (**Figures 3C, D**). Additionally, the application of 50 μM Tyr A23 blocked the Pep1 effects on PIN2-GFP internalization (**Figure 3E**; **Supplementary Figure 4**).

**Figure 4:**
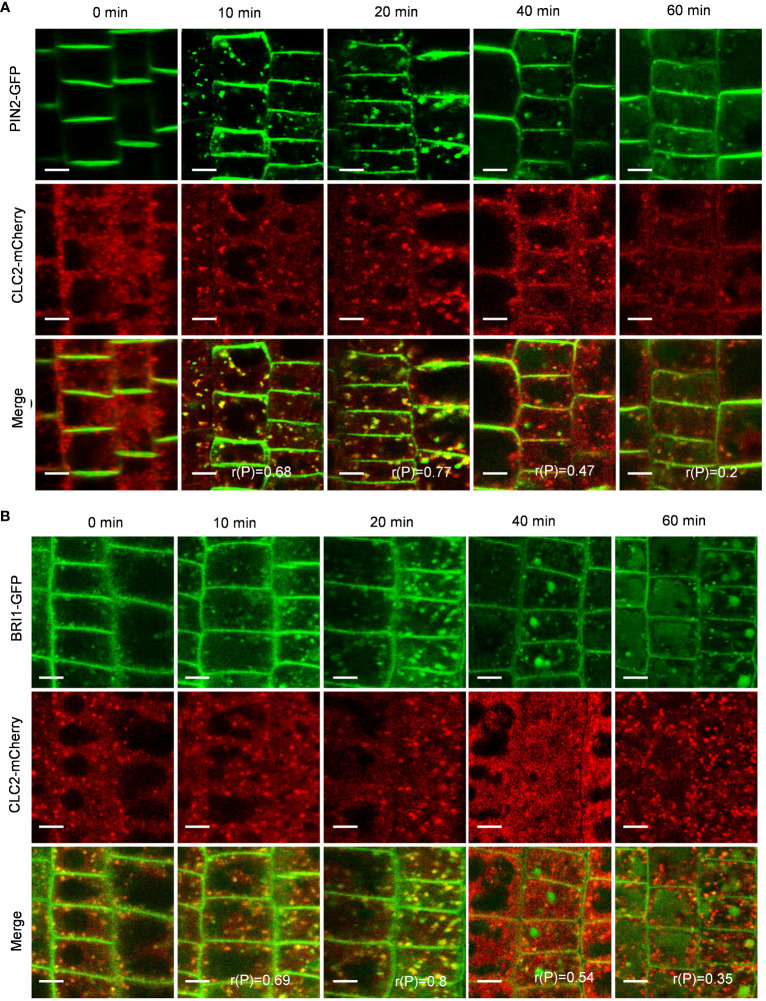
Clathrin dependence of Pep1-induced responses. **(A, B)** The colocalization analyze of PIN2-GFP **(A)** and BRI1-GFP **(B)** with CLC2-mCherry under Pep1 treatment. The roots of 6-d-old transgenic plants were stained in 2 μM FM4-64 solution for 5 min, rinsed three times, and incubated in half-strength MS liquid medium with 100 nM Pep1 as indicated for 10, 20, 40, and 60 min. Bars=5 μm. r(P) indicates the percentage of signal overlap, and an r(P) value of 1.0 represents 100% colocalization. Bars=5 μm.

In the CME pathway, clathrin invaginates and encases cargo to form clathrin-coated vesicles (CCVs), which subsequently undergo intracellular transport (Mettlen et al., 2018). To investigate whether the PIN2-GFP- and BRI1-GFP-labeled intracellular puncta were invaginated into CCVs, we quantified the colocalization of PIN2-GFP or BRI1-GFP with CLC2-mCherry. As expected, the Pep1-induced PIN2-GFP- and BRI1-GFP-labeled intracellular puncta exhibited high colocalization with CLC2-mCherry in CCVs (**Figures 4A, B**). The linear Pearson correlation coefficient (rP) for colocalization between PIN2-GFP or BRI1-GFP and CLC2-mCherry puncta exceeded 0.6 after 10- and 20- minutes chase of Pep1 treatment. These observations suggest that the PIN2 and BRI1 are internalized through CME pathway, and CLC2 and CLC3 are crucial for this process.

### Pep1 promotes PM internalization through TGN/EE and PVC pathways

In plants, the continuous endocytosis of PM cargo results in the accumulation of cargo in endosomes, where they can be either recycled back to the PM or targeted for degradation in the vacuole (Kaksonen and Roux, 2018; Mettlen et al., 2018; Aniento et al., 2022). The trans-Golgi network/early endosome (TGN/EE) acts as the first acceptor compartment for endocytosed proteins and serves as a sorting station for deciding whether proteins should be recycled or degraded (Zhang et al., 2019). However, it has been reported that the endomembrane trafficking of the Pep1-PEPRs complex operates independently of the TGN/EE pathway (Ortiz-Morea et al., 2016). Over an extended period of Pep1 treatment (40 and 60 minutes), PIN2-GFP and BRI1-GFP became concentrated in structures resembling vacuoles, coinciding with reductions in PM-localized and intracellular fluorescent signals. This suggests that PIN2-GFP and BRI1-GFP undergo degradation after Pep1 treatment (**Figures 1A–D**).

**Figure 5:**
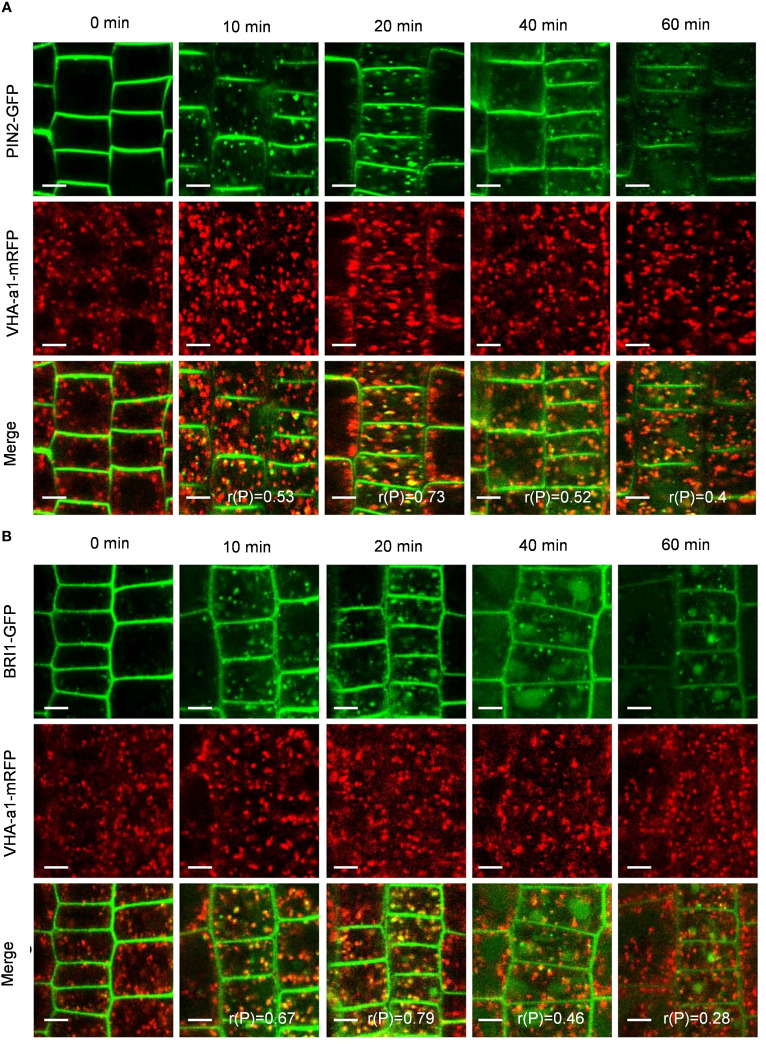
TGN/EE dependence of Pep1-induced PIN2-GFP and BRI1-GFP internalization. **(A, B)** The co-localization analysis of PIN2-GFP **(A)** and BRI1-GFP **(B)** with VHA-a1-mRFP under Pep1 treatment. The roots of 6-day-old transgenic plants were treated with 100 nM Pep1 as indicated for 10, 20, 40, and 60 min. Bars=5 μm. r(P) indicate the percentage of signal overlap, and an r(P) value of 1.0 represents 100% colocalization.

**Figure 6:**
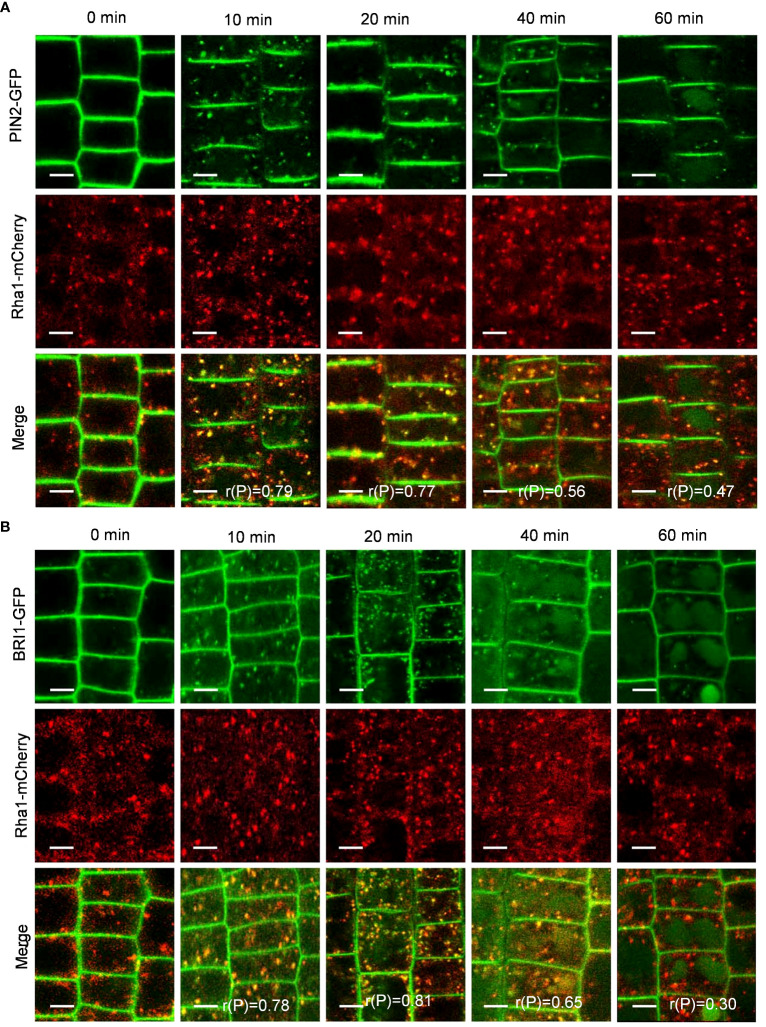
PVC dependence of Pep1-induced PIN2-GFP and BRI1-GFP internalization. **(A, B)** The colocalization analysis of PIN2-GFP **(A)** and BRI1-GFP **(B)** with Rha1-mCherry under Pep1 treatment. The roots of 6-day-old transgenic plants were treated with 100 nM Pep1 as indicated for 10, 20, 40, and 60 min. Bars=5 μm. r(P) indicate the percentage of signal overlap, and an r(P) value of 1.0 represents 100% colocalization.

To verify whether PIN2-GFP and BRI1-GFP are transported to vacuoles via the TGN/EE pathway, we examined the colocalization of PIN2-GFP and BRI1-GFP with VHA-a1-mRFP, a TGN/EE marker (Dettmer et al., 2006). Interestingly, the Pep1-induced intracellular puncta of PIN2-GFP and BRI1-GFP highly overlapped with VHA-a1-mRFP (**Figures 5A, B**), The linear (rP) value for colocalization of PIN2-GFP with VHA-a1-mRFP was 0.53 and 0.73 and BRI1-GFP with VHA-a1-mRFP exceeded 0.6 at 10- and 20- minutes chase of Pep1 treatment, respectively (**Figures 5A, B**), indicating that the TGN/EE component is crucial for the endomembrane trafficking of PIN2 and BRI1 during Pep1 treatment. Additionally, we observed that the intracellular puncta of PIN2-GFP and BRI1-GFP also colocalized with Rha1-mCherry, a prevacuolar compartment (PVC) marker (Wang et al., 2017) (**Figures 6A, B**). This suggests that the PVC pathway is required to mediate the Pep1-induced PM internalization. We ruled out the involvement of the Golgi apparatus pathway in the process of Pep1-induced PM internalization because the intracellular puncta of PIN2-GFP did not colocalized with the Golgi apparatus marker SYP32-mCherry (Geldner et al., 2009) (**Supplementary Figure 5**). Taken together, we have confirmed that the TGN/EE- and PVC-dependent trafficking pathways are involved in mediating the Pep1-induced endocytic degradation of PM proteins. The mechanism is distinct from the endocytosis of the Pep1-PEPRs complex, as reported previously (Ortiz-Morea et al., 2016).

### SA inhibits the effects of Peps on PM endocytosis

**Figure 7:**
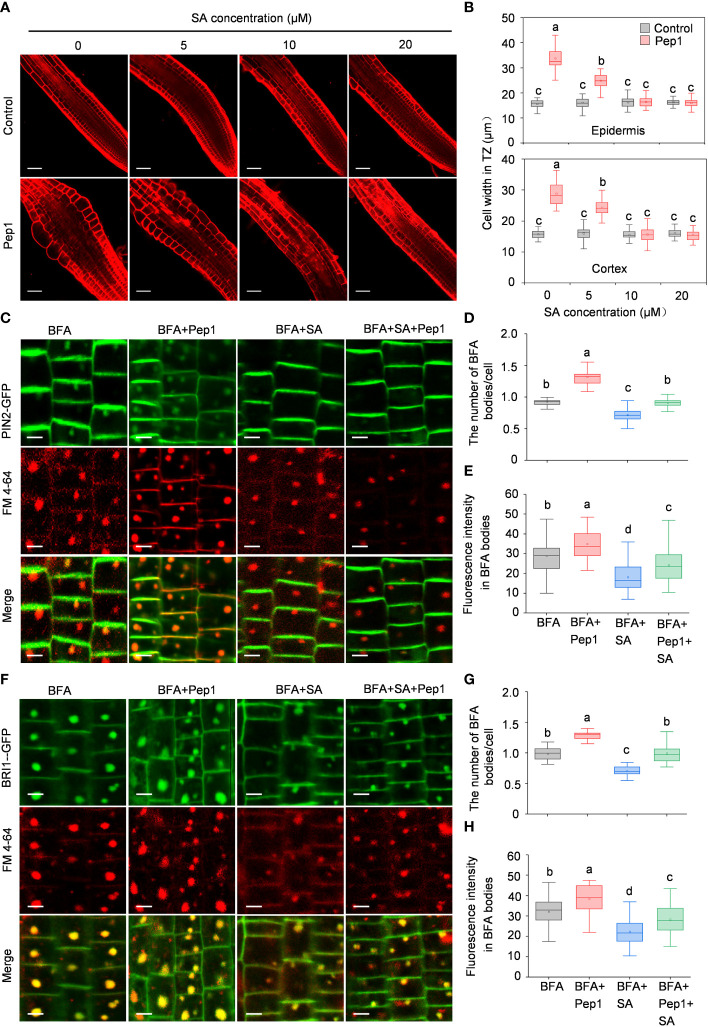
SA interferes the Pep1 responses. **(A)** The cell swelling in root transition zone. Five-day old WT seedings were transferred onto half-strength MS agar medium supplemented with or without (control) 100 nM Pep1 in the presence of SA (ranged from 0 to 50 μM) for 12 h. The roots were stained with 5 uM propidium iodide (PI) for 15 s and photographed. The experiments were repeated three times with similar results. Bars = 100 um. **(B)** Quantitative analysis of epidermal and cortex cell width in TZ as in **(A)** (n= 30 cells from 6 roots per treatment). Boxs with different letters indicate significant differences as defined by two-way ANOVA with Tukey’s test (p < 0.05). **(C)** Evaluation of the BFA-visualized internalization of PIN2-GFP. Bars=5 μm. **(D)** Quantification of the BFA-visualized internalization of PM proteins in PIN2-GFP as in **(C)** (n= 50 cells from 8 roots per treatment). **(E)** Quantification of the PIN2-GFP fluorescence intensity in BFA bodies as in **(C)**. (n= 100 cells from 10 roots per treatment). **(F)** Evaluation of the BFA-visualized internalization of BRI1-GFP. Bars=5 μm. **(G)** Quantification of the BFA-visualized internalization of PM proteins in BRI1-GFP as in **(F)** (n= 50 cells from 8 roots per treatment). **(H)** Quantification of the BRI1-GFP fluorescence intensity in BFA bodies as in **(F)** (n= 100 cells from 10 roots per treatment). The 6-day-old seedlings were treated with 25 μM BFA or 25 μM BFA cotreated with either 100 nM Pep1, 10 μM SA or 100 nM Pep1 + 10 μM SA for 60 min in **(C)** to **(H)**. In **(D, E, G, H)**, Boxs with different letters indicate significant differences as defined by one-way ANOVA with Tukey’s test (p < 0.05).

Salicylic acid (SA) has been reported to interfere with clathrin-mediated endocytic protein trafficking and negatively regulate plant hypersensitive responses during pathogen infections (Du et al., 2013; Radojicic et al., 2018; Zhang and Li, 2019). To investigate whether SA plays a role in regulating the Pep1’s effect in roots, we initially co-treated roots with various concentrations of SA (ranging from 5 to 20 μM) alongside 100 nM Pep1 to assess cell swelling. As depicted in **Figures 7A, B**, the application of SA dampened the Pep1-induced swelling, with 10 μM SA fully blocking the effects of Pep1 (**Figures 7A, B**). We proceeded to examine PM endocytosis under SA treatment. The use of 10 μM SA inhibited the BFA-induced internalization of PIN2-GFP and BRI1-GFP (**Figures 7C–H**), aligning with the established notion that SA interferes with endocytosis (Du et al., 2013). Furthermore, a noticeable change occurred when we co-treated roots with 10 μM SA and 100 nM Pep1 to analyze PM endocytosis. The application of SA suppressed the effect of Pep1 on the PIN2-GFP and BRI1-GFP fluorescence signals accumulation in BFA bodies (**Figures 7C–H**). In summary, these results indicate that SA negatively regulates the Pep1-induced PM endocytosis.

## Discussion

Plants defend themselves against pathogen attacks through highly conserved innate immune systems. The two well-known patterns, MAMPs and DAMPs, are recognized by related PRRs, activating plant PTI responses (Bartels and Boller, 2015). In *Arabidopsis*, the DAMPs, such as Pep1, combine with their receptor PEPRs and undergo endocytosis dependent on the CME pathway (Ortiz-Morea et al., 2016). Our previous study indicated that Pep1 treatment could also induce the endocytosis of PIN2 through an uncertain route (Jing et al., 2019). In this study, we used PM-localized PIN2 and BRI1 as markers to analyze the potential mechanism of Pep1’s effects on PM endocytosis. We demonstrated that Pep1 treatment induces the endocytosis of PIN2 and BRI1 through the CME pathway. The internalized cargo of PIN2 and BRI1 is transported to the vacuole through the TGN/EE and PVC pathways. Intriguingly, SA treatment suppresses the effect of Pep1 on PM endocytosis. These findings unveil a previously unrecognized signaling pathway by which danger peptides regulate PM dynamics.

A growing body of research shows that the dynamics of PM proteins through endocytosis play an essential role in regulating plant immunity in two ways. First, during pathogen infection, surface-localized plant immune receptors undergo endocytosis when interacting with related PAMPs or DAMPs, activating downstream immune responses. Second, certain eukaryotic pathogens, including oomycete and fungal pathogens, deliver their effector proteins into host cells to promote virulence through the endocytic pathway (Robatzek et al., 2006; Leborgne-Castel et al., 2008; Kale and Tyler, 2011; Gu et al., 2017). *Arabidopsis* Pep1 has been reported to combine with its receptor PEPR1 and undergo endocytosis in a clathrin-dependent manner (Ortiz-Morea et al., 2016). The internalized Pep1-PEPR1 cargo is subsequently transported to the vacuole and undergoes degradation (Ortiz-Morea et al., 2016). The internalization of Pep1-PEPR is likely a mechanism used to desensitize cells after Pep1 stimulation, similar to the mechanisms that involve BR-BRI1 and flg22-FLS2 endocytosis (Irani et al., 2012; Smith et al., 2014; Mbengue et al., 2016). In this study, we discovered that Pep1 treatment could also induce the endocytosis of other PM proteins, such as PIN2 and BRI1, in a CME-dependent manner. The two clathrin light chains, CLC2 and CLC3, play a vital role in regulating the effect of Pep1. The Pep1 induced internalization of FM4-64–labeled PM, as well as PM proteins PIN2 and BRI1 is a rapid process that occurred at 10 min time point, which displayed somewhat earlier compared with the previous results, the internalization of FM4-64–labeled PM and PEPR1-GFP induced by Pep1 occured at 20 min time point (Ortiz-Morea et al., 2016). We speculate that the cell status in root or the purity of Pep1 may have led to different outcomes. Even-though, the two results indicate that the Pep1-induced endocytosis of PEPRs, PIN2, and BRI1 is a rapid process that likely strongly triggers cell immune responses to defend against external pathogenic threats. However, it is currently unclear whether these small peptides induce the endocytosis of PM proteins is a general or specific process, which requires more in-depth research.

The internalized receptors and transporter cargo are gathered into TGN/EEs and further transported into the vacuole. In this study, we found the TGN/EE-localized vacuolar H^+^-ATPase is required to control the PIN2 and BRI1 internalization, which is different from the Pep1-PEPR trafficking pathway, where complexes are internalized directly into MVBs, bypassing the TGN/EE or are transported to the MVBs via a V-ATPase–negative subpopulation of the TGN (Ortiz-Morea et al., 2016). The reasons for these differences in trafficking routes are not clear at present, indicating a variety of responses induced by Pep1 to regulate cellular immunity, which warrants further in-depth research.

SA has long been known to play essential roles in regulating plant immunity. Upon pathogen infection, SA is synthesized through the isochorismate pathway and perceived by two groups of receptors, NPR1 and NPR3/NPR4, to regulate plant systemic acquired resistance (SAR), PAMPs-triggered immunity (PTI), and effector-triggered immunity (ETI) (Radojicic et al., 2018; Zhang and Li, 2019). In plants, PAMPs and pathogenic effector-induced immunogenic cell death are broadly termed hypersensitive response (HR), involving the generation of reactive oxygen species (ROS) and the elevation of intracellular Ca^2+^ levels (Pitsili et al., 2020; Saur et al., 2021). SA has been reported to negatively regulate ETI-induced HR, as a high level of SA or overexpression of NPR1 suppresses effector-induced cell death (Radojicic et al., 2018; Zhang and Li, 2019). We speculate that the negative regulatory effect of SA on Pep1 responses is similar to that of pathogenic effectors because Pep1 treatment also triggers significant immune responses and cell death (Jing et al., 2019). SA treatment suppressed the effect of Pep1 on promoting callose and lignin deposition (Jing et al., 2023), inducing cell swelling and PM protein internalization (**Figure 5**). The two SA-deficient mutants, *SA-deficient 2* (*sid2*) and *enhanced disease susceptibility 5* (*eds5*), were found to enhance the Pep1 effect (Jing et al., 2023). The negative regulatory effect of SA on Pep1 may have significant implications. Under pathogen infection, plants release Pep1 to activate immune responses, Pep1 disrupts cell membrane integrity by promoting PM protein degradation. The peptide further induces cell swelling, leading to cell death, a key step in HR to restrict pathogen proliferation. Once the plant overcomes the pathogen, continuous Pep1 presence in cells disrupts cell growth. At this stage, the high level of SA in the plant negatively regulates Pep1 signaling, suppresses cell death, and allows the plant to return to normal growth. However, the mechanism by which SA inhibits Pep1 is not clear and requires further study.

In conclusion, this study uncovers a significant role of Pep1 in PM endocytosis. Further investigation into the differences in endocytosis between Pep1-PEPR and other PM proteins will enhance our understanding of plant immunity and how plant elicitor peptides control PM dynamics to regulate plant immunity.

## Materials and methods

### Plant materials and growth conditions

*Arabidopsis* (*Arabidopsis thaliana*) T-DNA insertion lines, namely *clc2-1* (SALK_016049), *clc3-1* (CS100219), and *clc2-1 clc3-1* (Wang et al., 2013), as well as transgenic lines in Columbia-0 background, including PIN2-GFP (Blilou et al., 2005), BRI1-GFP (Geldner et al., 2007), CLC2-mCherry (Li et al., 2011), VHA-a1-mRFP (Dettmer et al., 2006), Rha1-mCherry (Wang et al., 2017), and SYP32-mCherry (Geldner et al., 2009) used in this study were described previously. The PIN2-GFP/*clc2 clc3* line was obtained through a cross between PIN2-GFP and *clc2 clc3* (Wang et al., 2013). Similarly, the PIN2-GFP/CLC2-mCherry, PIN2-GFP/VHA-a1-mRFP, PIN2-GFP/Rha1-mCherry, and PIN2-GFP/SYP32-mCherry materials were obtained by crossing PIN2-GFP with CLC2-mCherry, VHA-a1-mRFP, Rha1-mCherry, and SYP32-mCherry, respectively. Seedlings were cultivated on half-strength Murashige and Skoog (MS) medium containing 1% sucrose and 0.8% phytogel (Sigma-Aldrich, St. Louis, MO, USA). These growth conditions maintained a light intensity of 90 μmol/m^2^/s with a photoperiod of 16 hours light and 8 hours dark at 22°C.

### Peptide synthesis

The Pep1 peptides employed in this study were synthesized by GL Biochem, featuring the following amino acid sequence from the N-terminus to the C-terminus: ATKVKAKQRGKEKVSSGRPGQHN. The peptide was dissolved in water to form 1 mM stock solution. The stock solution was dissolved with half-strength MS liquid medium to prepare the desired concentration of the working solution.

### Cell swelling assay

Five-day-old seedlings were transplanted onto half-strength MS agar medium supplemented with various drugs for a 12-hour duration. Subsequently, the roots were stained with 5 μM propidium iodide (PI) for 15 seconds. The roots were mounted with sterile water and imaged using an LSM-710 argon/krypton laser scanning confocal microscope (Zeiss) with a 20 × objective. The excitation wavelengths for propidium iodide signals were set at 543 nm and emission was collected between 580 and 630 nm. Z-stack images were collected with 3 μm steps and the scan speed was 8 s/scan. The width of epidermis and cortex cells in the transition zone of the root was quantified using Image J software.

### Plasma membrane internalization assay

To assess plasma membrane internalization, the roots were immersed in a 2 μM FM 4-64 (Sigma-Aldrich, St. Louis, MO, USA) solution (dissolved in half-strength MS liquid medium) for 5 minutes, followed by three rinses. Subsequently, they were incubated in half-strength MS liquid medium with various chemical treatments for 10, 20, 40, and 60 minutes. The roots were mounted with sterile water and the cortex cells in the root meristem zone were captured using an LSM-710 argon/krypton laser scanning confocal microscope (Zeiss) with a 63× objective. For endocytosis signal analysis, the roots were treated with 25 μM BFA (Sigma-Aldrich, St. Louis, MO, USA) or 25 μM BFA co-treated with different chemicals for 60 minutes. The roots were photographed under LSM-710 confocal microscope. The GFP-labeled BFA bodies were quantified per cell. Also the GFP fluorescence signals in BFA bodies were quantified using Image J software. To quantitatively analyze GFP fluorescence intensity, confocal images were captured under strictly identical acquisition parameters, which included laser power, photomultiplier settings, offset, zoom factor, and resolution, across all experimental root samples. FM 4-64 excitation at 514 nm and emission at 600-700 nm. GFP signals were excited at 488 nm wavelength and collected emission between 495 and 550 nm. The cortex cells in root meristem zone were selected as regions of interest (ROI) to assay the PM internalization. Z-stack images were acquired from top to bottom of the cells with 1 μm steps and the scan speed was 8 s/scan. The linear Pearson correlation coefficient (rP) was employed with Image J software to indicate the degree of colocalization. The apical side of PM and the cytoplasmic region in ROI were selected with a brush tool in Image J software to analyze the PM and internal fluorescence signal, respectively.

### Statistical analysis

For co-localization analyses, 50 cells from 8 roots were analyzed in each of time point treatment. For cell swelling analyses, 30 cells from 6 roots were analyzed in each of treatment. For fluorescence intensity analyze in BFA bodies, 100 cells from 10 roots were analyzed in each of time point treatment. Each experiment was independently repeated three times. Statistical analysis was conducted using one-way or two-way ANOVA followed by Tukey’s test. Boxs with different letters indicate significant differences (p < 0.05).

## Data availability statement

The original contributions presented in the study are included in the article/**Supplementary Material**. Further inquiries can be directed to the corresponding authors.

## Author contributions

YJ: Data curation, Formal analysis, Funding acquisition, Writing – original draft, Writing – review & editing. XZ: Data curation, Formal analysis, Funding acquisition, Writing – original draft, Writing – review & editing. RS: Data curation, Writing – review & editing. JC: Data curation, Formal analysis, Writing – original draft, Writing – review & editing.

## Supplementary material

Supplementary Figure 1 Pep1 induces the internalization of PM proteins. **(A)** Six-day-old wild-type roots were stained in 2 μM FM4-64 solution for 5 min, rinsed three times, and incubated in half-strength MS liquid medium with or without (Control) 100 nM Pep1 as indicated for 10, 20, 40, and 60 min. The experiments were repeated three times with similar results. Bars=5 μm. **(B, C)** Quantitative analysis of plasma membrane **(B)** and intracellular fluorescence intensity **(C)** as in **(A)**. Data are means ± SD from three independent experiments (n= 50 cells from 8 roots per treatment). Asterisks in **(B, C)** indicate statistically significant differences compared to control at each of time point treatment (Tukey’s test, *P <0.05).

Supplementary Figure 2 Pep1 promotes the BFA-visualized internalization of PM proteins. **(A)** Quantification of the BFA-visualized internalization of PM proteins in PIN2-GFP (n= 50 cells from 8 roots per treatment). **(B)** Quantification of the PIN2-GFP fluorescence intensity in BFA bodies (n= 100 cells from 10 roots per treatment). **(C)** Quantification of the BFA-visualized internalization of PM proteins in BRI1-GFP (n= 50 cells from 8 roots per treatment). **(D)** Quantification of the BRI1-GFP fluorescence intensity in BFA bodies (n= 100 cells from 10 roots per treatment). Six-day-old seedlings were treated with 25 μM BFA or 25 μM BFA cotreated with either 100 nM Pep1, 50 μM CHX or 100 nM Pep1 + 50μM CHX for 60 min. In panels **(A–D)**, Boxs with different letters indicate significant differences as defined by one-way ANOVA with Tukey’s test (p < 0.05).

Supplementary Figure 3 Clathrin dependence of Pep1-induced cell swelling. **(A)** The cell swelling in root transition zone. Five-day-old WT and *clc2 clc3* seedings were transferred onto half-strength MS agar medium supplement with or without (Control) 100 nM Pep1, 50 μM Tyr A23 or 100 nM Pep1 + 50 μM Tyr A23 for 12 h. The roots were stained with 5 uM propidium iodide (PI) for 15 s and photographed under a confocal laser-scanning microscope. The experiments were repeated three times with similar results. Bars = 100 um. **(B, C)** Quantitative analysis of epidermal and cortex cell width in TZ as in **(A)** (n= 30 cells from 6 roots per treatment). Boxs with different letters indicate significant differences as defined by two-way ANOVA with Tukey’s test (p < 0.05).

Supplementary Figure 4 Clathrin dependence of Pep1-induced PIN2 internalization. **(A, B)** Quantification of the BFA-visualized internalization of PIN2-GFP **(A)** and PIN2-GFP fluorescence intensity in BFA bodies **(B)** in roots of wild type (WT) and *clc2 clc3* double mutant (n= 50 cells from 8 roots per treatment). Six-day-old seedlings were treated with 25 μM BFA or 25 μM BFA cotreated with either 100 nM Pep1, 100 nM Pep1 + 50 μM Tyr A23, 100 nM Pep1 + 50 μM CHX or 100 nM Pep1 + 50 μM Tyr A23 + 50 μM CHX for 60 min. Boxs with different letters indicate significant differences as defined by two-way ANOVA with Tukey’s test (p < 0.05).

Supplementary Figure 5 The trafficking of PIN2 induced by Pep1 independent with Golgi apparatus pathway. The co-localization analysis of PIN2-GFP with SYP32-mCherry under Pep1 treatment. The roots of 6-d-old transgenic plants were treated with 100 nM Pep1 as indicated for 10, 20, and 60 min. Boxs=5 μm. r(P) indicate the percentage of signal overlap, and an r(P) value of 1.0 represents 100% colocalization.
