# Supplementary material for: Plant elicitor peptide induces endocytosis of plasma membrane proteins in Arabidopsis
Source: Front Plant Sci. 2023 Dec 22;14:1328250. doi: 10.3389/fpls.2023.1328250 (PMC10766710; doi:10.3389/fpls.2023.1328250)
Supplement: Supplementary Figure 1 — Pep1 induces the internalization of PM proteins. (A) Six-day-old wild-type roots were stained in 2 μM FM4-64 solution for 5 min, rinsed three times, and incubated in half-strength MS liquid medium with or without (Control) 100 nM Pep1 as indicated for 10, 20, 40, and 60 min. The experiments were repeated three times with similar results. Bars=5 μm. (B, C) Quantitative analysis of plasma membrane (B) and intracellular fluorescence intensity (C) as in (A). Data are means ± SD from three independent experiments (n= 50 cells from 8 roots per treatment). Asterisks in (B, C) indicate statistically significant differences compared to control at each of time point treatment (Tukey’s test, *P <0.05). [file DataSheet_1.pdf]

Plant Elicitor Peptide Induces Endocytosis of Plasma Membrane Proteins in Arabidopsis

Supplementary Figures and Legends

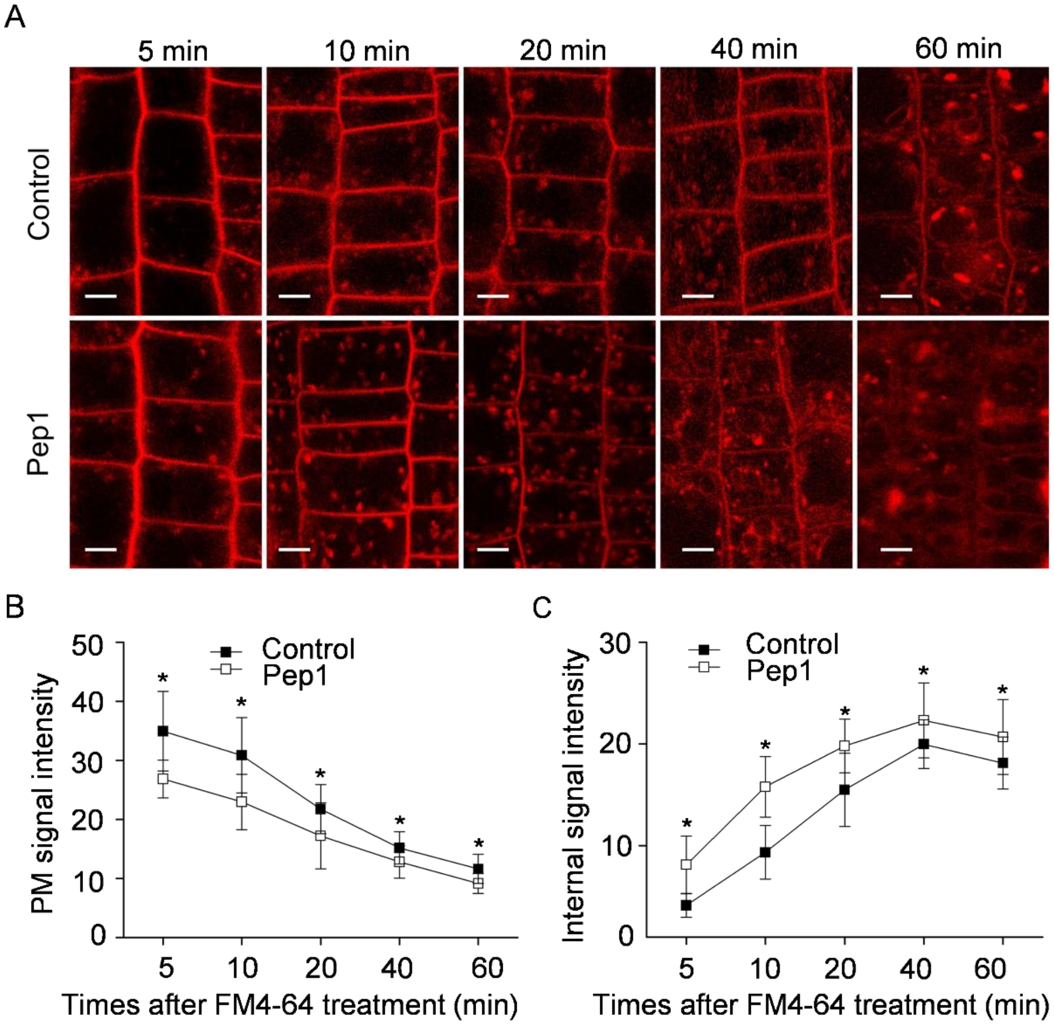

Supplementary figure 1 Pep1 induces the internalization of PM proteins. (A) Six-day-old wild-type roots were stained in 2 μM FM4-64 solution for 5 min, rinsed three times, and incubated in half-strength MS liquid medium with or without (Control) 100 nM Pep1 as indicated for 10, 20, 40, and 60 min. The experiments were repeated three times with similar results. Bars=5 μm. (B) and (C) Quantitative analysis of plasma membrane (B) and intracellular fluorescence intensity (C) as in (A). Data are means ±SD from three independent experiments (n= 50 cells from 8 roots per treatment). Asterisks in (B) and (C) indicate statistically significant differences compared to control at each of time point treatment (Tukey's test, *P <0.05).

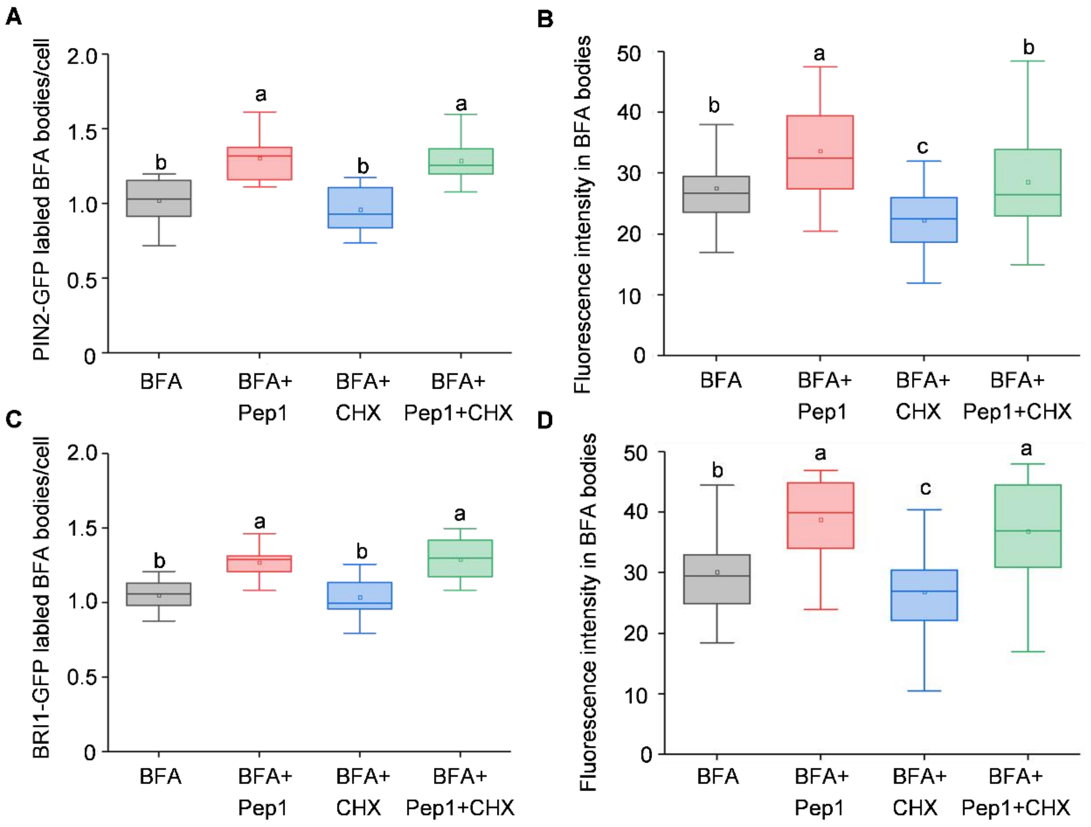

Supplementary figure 2. Pep1 promotes the BFA-visualized internalization of PM proteins. (A) Quantification of the BFA-visualized internalization of PM proteins in PIN2-GFP (n= 50 cells from 8 roots per treatment). (B) Quantification of the PIN2-GFP fluorescence intensity in BFA bodies (n= 100 cells from 10 roots per treatment). (C) Quantification of the BFA-visualized internalization of PM proteins in BRI1-GFP (n= 50 cells from 8 roots per treatment). (D) Quantification of the BRI1-GFP fluorescence intensity in BFA bodies (n= 100 cells from 10 roots per treatment). Six-day-old seedlings were treated with 25 μM BFA or 25 μM BFA cotreated with either 100 nM Pep1, 50 μM CHX or 100 nM Pep1+50μM CHX for 60 min. In panels (A) to (D), Boxs with different letters indicate significant differences as defined by one-way ANOVA with Tukey's test ($p < 0.05$).

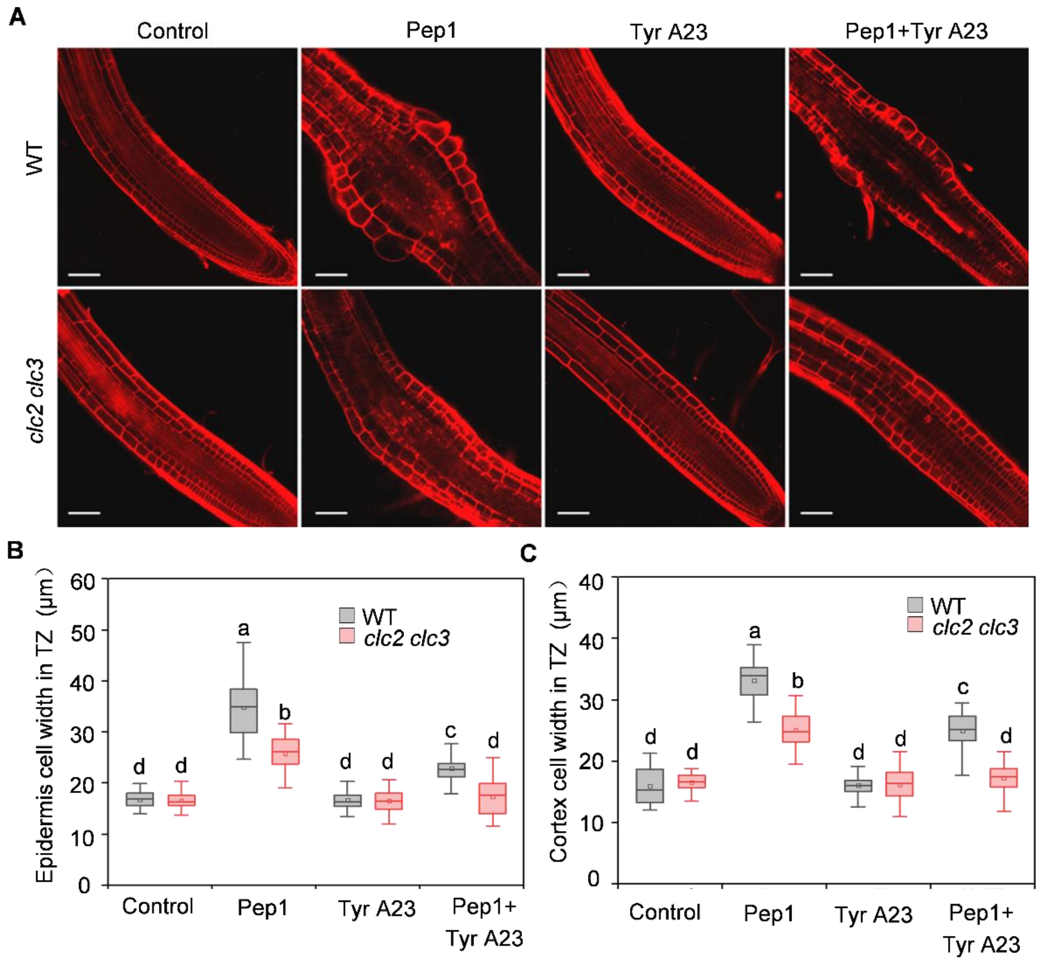

Supplementary figure 3. Clathrin dependence of Pep1-induced cell swelling. (A) The cell swelling in root transition zone. Five-day-old WT and *clc2 clc3* seedings were transferred onto half-strength MS agar medium supplement with or without (Control) 100 nM Pep1, 50 μM Tyr A23 or 100 nM Pep1 + 50 μM Tyr A23 for 12 h. The roots were stained with 5 uM propidium iodide (PI) for 15 s and photographed under a confocal laser-scanning microscope. The experiments were repeated three times with similar results. Bars = 100 um. (B) and (C) Quantitative analysis of epidermal and cortex cell width in TZ as in (A) (n= 30 cells from 6 roots per treatment). Boxs with different letters indicate significant differences as defined by two-way ANOVA with Tukey's test ($p < 0.05$).

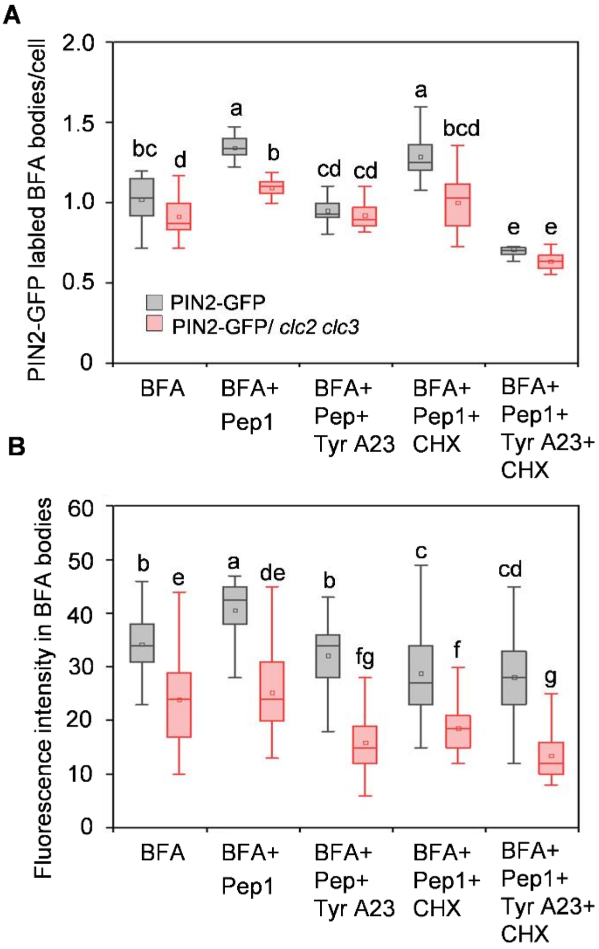

Supplementary figure 4. Clathrin dependence of Pep1-induced PIN2 internalization.

(A) and (B) Quantification of the BFA-visualized internalization of PIN2-GFP (A) and PIN2-GFP fluorescence intensity in BFA bodies (B) in roots of wild type (WT) and *clc2 clc3* double mutant (n= 50 cells from 8 roots per treatment). Six-day-old seedlings were treated with 25 μM BFA or 25 μM BFA cotreated with either 100 nM Pep1, 100 nM Pep1 + 50 μM Tyr A23, 100 nM Pep1 + 50 μM CHX or 100 nM Pep1 + 50 μM Tyr A23 + 50 μM CHX for 60 min. Boxs with different letters indicate significant differences as defined by two-way ANOVA with Tukey's test ($p < 0.05$).

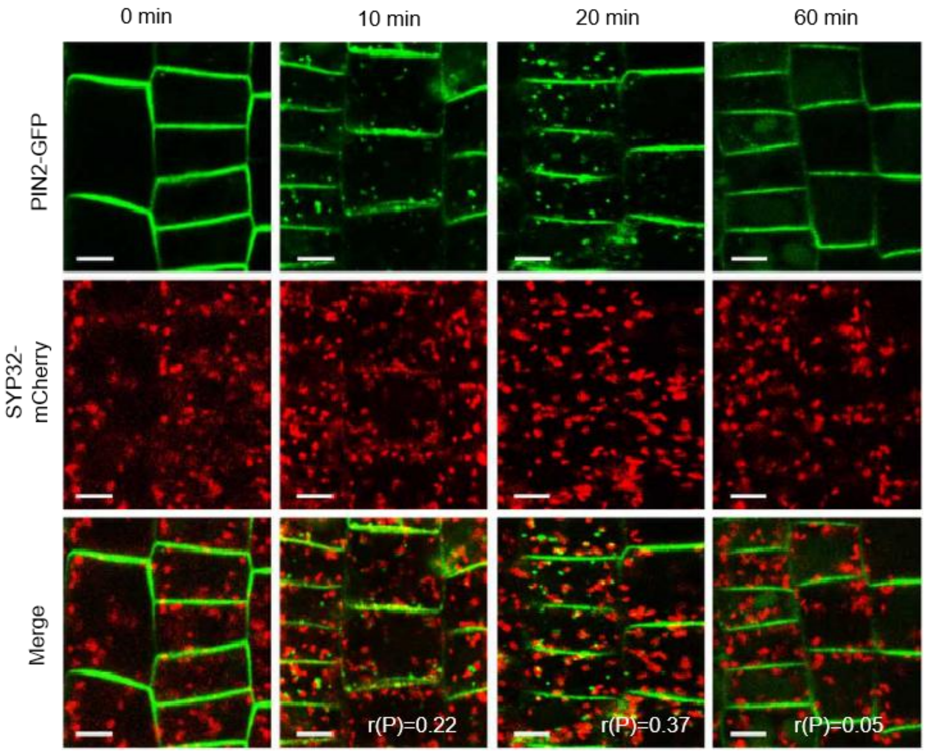

Supplementary figure 5. The trafficking of PIN2 induced by Pep1 independent with Golgi apparatus pathway. The co-localization analysis of PIN2-GFP with SYP32-mCherry under Pep1 treatment. The roots of 6-d-old transgenic plants were treated with 100 nM Pep1 as indicated for 10, 20, and 60 min. Bars=5 μm. r(P) indicate the percentage of signal overlap, and an r(P) value of 1.0 represents 100% colocalization.
